# Supplementary material for: An improved Bayesian Modified-EWMA location chart and its applications in mechanical and sport industry
Source: PLoS One. 2020 Feb 26;15(2):e0229422. doi: 10.1371/journal.pone.0229422 (PMC7043768; doi:10.1371/journal.pone.0229422)
Supplement: S1 File — (DOCX) [file pone.0229422.s001.docx]

**An improved Bayesian Modified-EWMA location chart and its applications in mechanical and sport industry**

**Muhammad Aslam^1^, ^*^Syed Masroor Anwar^1^**

**^1^**Department of Mathematics and Statistics, Riphah International University, 45210, Pakistan.

**^*^**Corresponding Author: masroorstatistics@gmail.com

**An Improved Bayesian Modified-EWMA Location Chart and its Applications in Mechanical and Sport Industry**

**Abstract**

Control charts are popular tools in the statistical process control toolkit and the exponentially weighted moving average (EWMA) chart is one of its essential component for efficient process monitoring. In the present study, a new Bayesian Modified-EWMA chart is proposed for the monitoring of the location parameter in a process. Four various loss functions and a conjugate prior distribution are used in this study. The average run length is used as a performance evaluation tool for the proposed chart and its counterparts. The results advocate that the proposed chart performs very well for the monitoring of small to moderate shifts in the process and beats the existing counterparts. The significance of the proposed scheme has proved through two real-life examples: (1) For the monitoring of the reaming process which is used in the mechanical industry. (2) For the monitoring of golf ball performance in the sports industry.

**Keywords:** Bayesian Framework; Conjugate Prior; Manufacturing Process; Normal Distribution

**1: Introduction**

Statistical process control (SPC) is an important and powerful technique for the enhancement of the product and process quality. The control charts are the familiar tool used within SPC to detect the out of control situations and hence monitor variation in a process [[1](#_ENREF_1)]. Shewhart [[2](#_ENREF_2)] introduced the basic control chart named Shewhart control chart for the monitoring of the process. The Shewhart charts are widely used to detect changes in the quality of the process due to it is simple to use without any substantial statistical pieces of training [[3](#_ENREF_3)]. Practically, these control charts are not very effective for the monitoring of the process when a small shift occurs in the process [[4](#_ENREF_4), [5](#_ENREF_5)].

This limitation of the Shewhart control chart is that it considers information from the current data and ignores the previous information contained in the data sequence [[6](#_ENREF_6)].

The exponentially weighted moving average (EWMA) charts are widely used for the monitoring of the small shift in the process as compared to the Shewhart charts [[7](#_ENREF_7)]. The EWMA charts accumulate current information as well as previous information and detect a small change in the process. The EWMA charts are extensively used and have many applications in the field of process monitoring. Various contributions are made to the literature by many authors, for example [[8](#_ENREF_8), [9](#_ENREF_9), [10](#_ENREF_10), [11](#_ENREF_11), [12](#_ENREF_12), [13](#_ENREF_13), [14](#_ENREF_14), [15](#_ENREF_15), [16](#_ENREF_16), [17](#_ENREF_17), [18](#_ENREF_18)].

The normal distribution is a very common and important distribution in Statistics. It is commonly used in the social and natural phenomena. Due to special nice properties, it has attracted a lot the attention of the researcher belongs to different fields. For example, Ajadi *et al.* [[19](#_ENREF_19)] suggested a control chart for the efficient monitoring of diabetic data under the assumption of normal distribution. Quinino *et al.* [[20](#_ENREF_20)] considered automobile engine piston ring data for the monitoring of the normal process. Chen and Chen [[21](#_ENREF_21)] investigated the injury of truck drivers using a mixed logit model. Chen *at al.* [[22](#_ENREF_22)] used a bivariate ordered probit model for the investigation of severe injuries of car drivers.

In the current years, the use of the Bayesian methods is very familiar in every field of science including engineering, metrology, demography, medical science, environmental science and quality control [[23](#_ENREF_23)]. For example, Zeng et al. [[24](#_ENREF_24)] proposed a spatial Tobit model using Bayesian methodology for the analysis of the crash rate on the roadside. Zeng et al. [[25](#_ENREF_25)] used a Bayesian approach for the analysis of multivariate Spatio-temporal Tobit regression. For more application-based study see [[26](#_ENREF_26),[27](#_ENREF_27)]. The Bayesian techniques are extensively used in the SPC and evaluation of various control charts. These techniques are utilized the current sample information as well as the prior knowledge that address the parameter’s uncertainty more precisely. For example, Girshick and Rubin [[28](#_ENREF_28)] developed control charts based on Bayesian methodology. Biswas [[29](#_ENREF_29)] and Montgomery [[30](#_ENREF_30)] developed Bayesian sampling plans for attributes. Later, Brush [[31](#_ENREF_31)] compared sampling plans in the presence of Bayesian and classical settings. Sharma and Bhuttani [[32](#_ENREF_32)] made a comparison of classical and Bayes consumer‘s risk. Menzefricke [[33](#_ENREF_33)] and Menzefricke [[34](#_ENREF_34)] proposed two control charts for the process mean and variance under the assumption of the Bayesian approach. Later, Saghir [[35](#_ENREF_35)] introduced the Bayesian mean chart for improved process monitoring. Recently, Menzefricke [[36](#_ENREF_36)] used the Bayesian approach and proposed the EWMA chart for the mean and variance of Normal distribution. Menzefricke [[37](#_ENREF_37)] also designed the Bayesian chart for location and dispersion of Normal distribution. For a more detailed study of Bayesian control charts, see [[23](#_ENREF_23),[38](#_ENREF_38)].

The role of loss functions is substantial in the Bayesian inference. Almost, all quality control (QC) researchers used loss functions for the process monitoring of Bayesian control charts. For example, Wu and Tian [[39](#_ENREF_39)] used weighted loss function (WLF) for the monitoring of the process location and dispersion shift in the CUSUM chart. Serel [[40](#_ENREF_40)] designed an EWMA chart for different linear, exponential and quadratic loss functions. Riaz and Ali [[41](#_ENREF_41)] employed various loss functions for the monitoring of the mean chart. For the Bayesian EWMA chart, three various symmetric and asymmetric loss functions are used by [[18](#_ENREF_18)]. The more detailed study of utilization of loss functions in the control charts is provided by [[42](#_ENREF_42), [43](#_ENREF_43), [44](#_ENREF_44)].

A Modified-EWMA chart is used for efficient monitoring of the process location [[45](#_ENREF_45)]. The Modified-EWMA chart detects a quick shift in the process as compared to the classical EWMA chart when an extra parameter is introduced in the model [[45](#_ENREF_45)]. Aslam *et al.* [[17](#_ENREF_17)] used Modified-EWMA for the COM-Poisson distribution. Anwar et al. [[46](#_ENREF_46)] proposed a Modified-mxEWMA chart for the process location in the presence of auxiliary information. A dispersion control chart with a Modified-EWMA statistic is proposed by [[47](#_ENREF_47)].

Riaz and Ali [[41](#_ENREF_41)] constructed a Bayesian Shewhart chart for the monitoring of a large shift in the location parameter. Similarly, Riaz *et al.* [[18](#_ENREF_18)] developed a Bayesian EWMA chart for the efficient monitoring of a small shift in the location parameter.

Because of the wide applicability of the Bayesian methodology in the quality control charts, in this article, we suggest a new Bayesian Modified-EWMA control chart for the process mean using various loss functions. The main objective of this paper is to purpose the Bayesian Modified-EWMA chart by combining the features of the existing Bayesian EWMA chart with the Modified-EWMA procedure for the improved process monitoring. Actually, we reformed and improved the Bayesian EWMA chart by Riaz *et al.* [[18](#_ENREF_18)] by introducing the Modified-EWMA statistic based on the posterior predictive distribution.

The remainder of the article is arranged as follows. In section 2, we explain a detailed study of the Bayesian approach and loss functions. In section 3, we discuss the Bayesian Modified-EWMA chart using various loss functions. The performance comparisons of the proposed chart are discussed in section 4. The last section is made for conclusions and recommendations.

**2: Bayesian approach**

A Bayesian approach is an alternative approach to the frequentist (classical) approach. Here, the parameter is considered as a "random variable" and is assumed to follow a prior distribution having certain parameters, known as hyperparameters. Two types of prior distributions are used to construct the posterior distribution, known as non-informative and informative priors. The Jeffreys and uniforms priors are commonly used non-informative priors and conjugate prior is commonly used as a family of informative prior [[48](#_ENREF_48)].

The posterior distribution is constructed by combing the sampling distribution (likelihood function) and prior distribution. Hence the posterior distribution is proportional to the product of the prior distribution and likelihood function. The posterior distribution for unknown parameter $\mu$ given $\boldsymbol{y}$ is defined by

$g\left( \mu|\mathbf{y} \right)=\frac{f\left( \mu\right)L\left( \cdot\right)}{\int_{-\infty}^{\infty} f\left( \mu\right)L\left( \cdot\right)d\mu}$ (1)

where $f\left( \mu\right)$ is the prior distribution, $L\left( \cdot\right)$ is the likelihood function, and $g\left( \mu|\mathbf{y} \right)$ is the posterior distribution for the parameter. The premium advantage of using the Bayesian approach is that it is naturally suitable for prediction of the future value. The posterior predictive distribution for the future variable $X$ given $Y$ is defined by

$g\left( x|\mathbf{y} \right)=\int_{-\infty}^{\infty} f\left( x|\mu\right)g\left( \mu| \boldsymbol{y} \right)d\mu$ (2)

**2.1: Loss Functions**

Loss Functions have a significant role in Bayesian inference. The loss function measures the losses produced by an erroneous estimation of a parameter. To obtain the best estimates in decision theory, a loss function must be specified. The loss functions may be symmetric or asymmetric. We have used the following loss functions in our study.

The Square Error loss function (SELF) is a symmetric loss function and extensively used loss function in Bayesian inference. If $X$ is predictive variable and $\mu_{SELF}$ is its estimate than SELF is defined as $L\left( X,\mu_{SELF} \right)=\left( X-\mu_{SELF} \right)^{2}$. The estimator $\mu_{SELF}$ which minimizes $L\left( X,\mu_{SELF} \right)$ is given by $\mu_{SELF}=E\left( X|\mathbf{y} \right)$ (3)

Varian [[49](#_ENREF_49)] proposed asymmetric loss function known as LINEX loss function (LLF), which is used in the case when overestimation is more significant. It is defined as $L\left( X,\mu_{LSELF} \right) =\exp\left\{ c\left( \mu_{LLF}-\mu\right) \right\}-c\left( \mu_{LLF}-\mu\right)-1,$ where $c\neq0$. When *c* is greater than zero, it is the case of overestimation. The predictive Bayes estimator of $X$ under LLF is

$\mu_{LLF}=-c^{-1}\ln\left[ E\left\{ exp(-c\left( X | \boldsymbol{y} \right)) \right\} \right]$ (4)

Rodrigues [[50](#_ENREF_50)] introduced Weighted Balance Loss function (WBLF) is widely used in the Bayesian Inference can be defined as $L\left( X,\mu_{WBLF} \right)=\left( \frac{X-\mu_{WBLF}}{\mu_{WBLF}} \right)^{2}$. The predictive Bayes estimator of $X$ which minimizes $L\left( X,\mu_{WBLF} \right)$ is given as

$\mu_{WBLF}=\frac{E\left( X^{2}|\mathbf{y} \right)}{E\left( X|\mathbf{y} \right)}$ (5)

The Precautionary Loss Function (PLF) was introduced by Norstrøm [[51](#_ENREF_51)]. When we are interested in study the low failure rate, then PLF is used to prevent underestimation. This is asymmetric loss function and easy to handle. PLF can be written as $L\left( X,\mu_{PLF} \right)=\frac{\left( X-\mu_{PLF} \right)^{2}}{\mu_{PLF}}$. The predictive Bayes estimator of $X$ under PLF case is

${\mu_{PLF}=\left\{ E\left( X^{2}|\mathbf{y} \right) \right\}}^{\frac{1}{2}}$ (6)

**3: The Proposed Monitoring Scheme**

In the following subsections, the proposed scheme is explained, followed by some existing control charts related to this era.

**3.1:** **Classical Modified-EWMA Chart**

Khan *et al.*[[45](#_ENREF_45)] introduced a Modified-EWMA Chart for the process mean. **S**uppose that the quality characteristic $Y$ belongs to the normal distribution with mean $\mu$ and variance $\sigma^{2}$. At the time (or subgroup) $t$, a random sample of size $n$ is taken and measure its quality characteristic. Estimating the following Modified-EWMA statistic at subgroup $t$, expressed by $X_{t}$.

$X_{t}=\lambda\bar{Y}_{t}+\left( 1-\lambda\right)X_{t-1}+\psi\left( \bar{Y}_{t}-\bar{Y}_{t-1} \right),$ (7)

here $\lambda\in\left( 0,1 \right)$ is the smoothing constant and $\psi$ is an additional constant concerned with the Modified-EWMA statistic. By continuous substitution $X_{t-i}$, the simplified form of Modified-EWMA statistic is given by

$X_{t}=\left( \lambda+\psi\right)\sum_{i=1}^{t-1} \left( 1-\lambda\right)^{i}\bar{Y}_{t-i}+\left( 1-\lambda\right)^{t}X_{0}+\psi\sum_{i=1}^{t-1} \left( 1-\lambda\right)^{i}\bar{Y}_{t-i-1}$ (8)

here, $X_{0}$ is assumed to be equal to $\mu_{0}$ (i.e. in-control process mean). The mean and variance of the Modified-EWMA statistic given in Equation (7) can be written as $E\left( X_{t} \right)=\mu_{0}$ and $Var\left( X_{t} \right)=\frac{\left( \lambda+2\psi\lambda+2\psi^{2} \right)}{n\left( 2-\lambda\right)}\sigma^{2}$. The time-varying limits of existing Modified-EWMA chart are

$\left. \begin{matrix} {LCL}_{t}=\mu_{0}-L\left[ \frac{\left( \lambda+2\psi\lambda+2\psi^{2} \right)\sigma^{2}}{n\left( 2-\lambda\right)}\left\{ 1-\left( 1-\lambda\right)^{2t} \right\} \right]^{\frac{1}{2}} \\ {UCL}_{t}=\mu_{0}+L\left[ \frac{\left( \lambda+2\psi\lambda+2\psi^{2} \right)\sigma^{2}}{n\left( 2-\lambda\right)}\left\{ 1-\left( 1-\lambda\right)^{2t} \right\} \right]^{\frac{1}{2}} \end{matrix} \right\}$ (9)

where $L$ is the coefficient of control chart, and $UCL$ and $LCL$ are upper and lower control limits. The value $\psi$ may be independent of $\lambda$, but according to Khan *et al.* [[45](#_ENREF_45)], $\psi$ is derived by minimizing the$Var\left( X_{t} \right)$, i.e. $\psi=-\frac{\lambda}{2}$. Also, for $\psi=0$, the Modified-EWMA chart is reduced to the classical EWMA chart.

**3.2: Proposed Bayesian Modified-EWMA Chart Under Various Loss Functions**

**S**uppose that quality characteristic $Y$ belongs to the normal distribution with mean $\mu$ and variance $\sigma^{2}$.i.e.

$g\left( y;\mu,\sigma^{2} \right)=\frac{1}{\sqrt{2\pi\sigma^{2}}}exp\left\{ -\frac{1}{{2\sigma}^{2}}\left( y-\mu\right)^{2} \right\}; -\infty\leq y,\mu\leq\infty,\sigma^{2}>0$ (10)

The likelihood function for a random sample of size $n$ is given by

$L\left( \boldsymbol{y} | \mu,\sigma^{2} \right)=\left( 2\pi\sigma^{2} \right)^{-\frac{n}{2}}exp\left\{ -\frac{1}{{2\sigma}^{2}}\sum_{i=1}^{n} \left( y_{i}-\mu\right)^{2} \right\}$ (11)

We assumed the informative prior for the parameter $\mu$, which is also a normal distribution with mean $a$ and variance $b^{2}$, i.e

. $g\left( \mu;a,b^{2} \right)=\frac{1}{\sqrt{2\pi b^{2}}}exp\left\{ -\frac{1}{{2b}^{2}}\left( \mu-a \right)^{2} \right\}; -\infty\leq\mu,a\leq\infty,b^{2}>0$ (12)

The prior distribution and the likelihood function are combined by using Equation (1), to construct the posterior distribution given as

$g\left( \mu| \boldsymbol{y} \right)=\frac{1}{\sqrt{2\pi\sigma_{n}^{2}}}exp\left\{ -\frac{1}{2\sigma_{n}^{2}}\left( \mu-\mu_{n} \right)^{2} \right\}; -\infty\leq\mu,\mu_{n}\leq\infty,\sigma_{n}^{2}>0$ (13)

where $\mu_{n}=\frac{a\sigma^{2}+nb^{2}\bar{Y}}{\sigma^{2}+nb^{2}}$ and $\sigma_{n}^{2}=\frac{b^{2}\sigma^{2}}{\sigma^{2}+nb^{2}}$. Now, the posterior predictive distribution is obtained, by using Equation (2) given as

$g\left( x | \boldsymbol{y} \right)=\frac{1}{\sqrt{2\pi\sigma_{X}^{2}}}exp\left\{ -{\frac{1}{2\sigma_{X}^{2}}\left( x-\mu_{n} \right)}^{2} \right\}; -\infty\leq x,\mu_{n}\leq\infty,\sigma_{X}^{2}>0$ (14)

where $\sigma_{X}^{2}=\sigma^{2}+\sigma_{n}^{2}$. Note that, the distribution of the predictive mean $\bar{X}|\boldsymbol{y}$ is also normal with mean $\mu_{n}$ and variance $\sigma_{X}^{2}=\frac{\sigma^{2}}{n}+\sigma_{n}^{2}$.

Based on the existing Modified-EWMA statistic given above, now we introduce the Bayesian Modified-EWMA chart by inserting the predictive mean $\left. \bar{X}_{t} \right|\boldsymbol{y}$ instead of the sample mean $\bar{Y}_{t}$ in the Modified-EWMA statistic given in (7). Hence, the Bayesian Modified-EWMA statistic is defined by

$Z_{t}=\lambda\left( \left. \bar{X}_{t} \right|\boldsymbol{y} \right)+\left( 1-\lambda\right)Z_{t-1}+\psi\left\{ \left( \left. \bar{X}_{t} \right|\boldsymbol{y} \right)-\left( \left. \bar{X}_{t-1} \right|\boldsymbol{y} \right) \right\}$ (15)

here $\lambda\in\left( 0,1 \right)$ is the smoothing constant and $\psi$ is an additional constant concerned with the proposed Bayesian Modified-EWMA statistic. The proposed Bayesian Modified-EWMA chart is reduced to the Bayesian EWMA chart by Riaz *et al.* [[18](#_ENREF_18)] for $\psi=0$. Here, $Z_{t}$ is the plotting statistic, which is updated with the predictive estimator $\left. \bar{X}_{t} \right|\boldsymbol{y}$. For the Bayesian Modified-EWMA chart, we discuss it under different loss functions given as.

*3.2.1: Bayesian Modified-EWMA under SELF*

Based on the (14), the posterior predictive estimator under SELF, using (3) is written as $\mu_{SELF}=\frac{a\sigma^{2}+nb^{2}\bar{Y}}{\sigma^{2}+nb^{2}}$. Under SELF, the posterior predictive time-varying control limits using Bayesian Modified-EWMA statistic in (15) are defined as

$\left. \begin{matrix} {LCL}_{t}=\mu_{SELF}-L\left[ \frac{\left( \lambda+2\psi\lambda+2\psi^{2} \right)\sigma_{X}^{2}}{n\left( 2-\lambda\right)}\left\{ 1-\left( 1-\lambda\right)^{2t} \right\} \right]^{\frac{1}{2}} \\ {UCL}_{t}=\mu_{SELF}+L\left[ \frac{\left( \lambda+2\psi\lambda+2\psi^{2} \right)\sigma_{X}^{2}}{n\left( 2-\lambda\right)}\left\{ 1-\left( 1-\lambda\right)^{2t} \right\} \right]^{\frac{1}{2}} \end{matrix} \right\}$ (16)

**3.2.2: Bayesian Modified-EWMA under LLF**

For LLF, the posterior predictive estimator under LLF using (4) is $\mu_{LLF}=\frac{a\sigma^{2}+nb^{2}\bar{Y}}{\sigma^{2}+nb^{2}}-\frac{c}{2}\left( \sigma^{2}+\frac{b^{2}\sigma^{2}}{\sigma^{2}+nb^{2}} \right)$. The posterior predictive time-varying control limits under LLF using Bayesian Modified-EWMA statistic in (15) are written as

$\left. \begin{matrix} {LCL}_{t}=\mu_{LLF}-L\left[ \frac{\left( \lambda\xi+2\psi\lambda+2\psi^{2} \right)\sigma_{X}^{2}}{n\left( 2-\lambda\right)}\left\{ 1-\left( 1-\lambda\right)^{2t} \right\} \right]^{\frac{1}{2}} \\ {UCL}_{t}=\mu_{LLF}+L\left[ \frac{\left( \lambda\xi+2\psi\lambda+2\psi^{2} \right)\sigma_{X}^{2}}{n\left( 2-\lambda\right)}\left\{ 1-\left( 1-\lambda\right)^{2t} \right\} \right]^{\frac{1}{2}} \end{matrix} \right\}$ (17)

**3.2.3 Bayesian Modified-EWMA under WBLF**

The posterior predictive estimator under WBLF using (5) is written as $\mu_{WBLF}=\frac{\left( nb^{2}\bar{Y}+a\sigma^{2} \right)^{2}+\left( \sigma^{2}+nb^{2} \right)\left( nb^{2}\sigma^{2}+b^{2}\sigma^{2}+\sigma^{4} \right)}{\left( \sigma^{2}+nb^{2} \right)\left( nb^{2}\bar{Y}+a\sigma^{2} \right)}$. Under WBLF, the posterior predictive time-varying control limits using Bayesian Modified-EWMA statistic in (15) are written as

$\left. \begin{matrix} {LCL}_{t}=\mu_{WBLF}-L\left[ \frac{\left( \lambda+2\psi\lambda+2\psi^{2} \right)\sigma_{X}^{2}}{n\left( 2-\lambda\right)}\left\{ 1-\left( 1-\lambda\right)^{2t} \right\} \right]^{\frac{1}{2}} \\ {UCL}_{t}=\mu_{WBLF}+L\left[ \frac{\left( \lambda+2\psi\lambda+2\psi^{2} \right)\sigma_{X}^{2}}{n\left( 2-\lambda\right)}\left\{ 1-\left( 1-\lambda\right)^{2t} \right\} \right]^{\frac{1}{2}} \end{matrix} \right\}$ (18)

**3.2.4: Bayesian Modified-EWMA under PLF**

For PLF, the posterior predictive estimator is ${\mu_{PLF}=\left\{ \frac{\left( nb^{2}\bar{Y}+a\sigma^{2} \right)^{2}+\left( \sigma^{2}+nb^{2} \right)\left( nb^{2}\sigma^{2}+b^{2}\sigma^{2}+\sigma^{4} \right)}{\left( \sigma^{2}+nb^{2} \right)^{2}} \right\}}^{\frac{1}{2}}$. The posterior predictive time-varying control limits using Bayesian Modified-EWMA statistic under PLF are presented as

$\left. \begin{matrix} {LCL}_{t}=\mu_{PLF}-L\left[ \frac{\left( \lambda+2\psi\lambda+2\psi^{2} \right)\sigma_{X}^{2}}{n\left( 2-\lambda\right)}\left\{ 1-\left( 1-\lambda\right)^{2t} \right\} \right]^{\frac{1}{2}} \\ {UCL}_{t}=\mu_{PLF}+L\left[ \frac{\left( \lambda+2\psi\lambda+2\psi^{2} \right)\sigma_{X}^{2}}{n\left( 2-\lambda\right)}\left\{ 1-\left( 1-\lambda\right)^{2t} \right\} \right]^{\frac{1}{2}} \end{matrix} \right\}$ (19)

**4: Performance Evaluation and Comparisons**

In this section, we used the Monte Carlo Simulation with 10,000 iterations to compute the average run length (ARL) of the Bayesian Modified-EWMA chart. In this regard, we considered various values of smoothing constant (0.05, 0.15, 0.30 and 0.70) and different sample sizes (5, 10 and 20). The $ARL_{0}$ and $ARL_{1}$ are the in-control (IC) and out-of-control (OC) ARL, respectively. A control chart with smaller ${ARL}_{1}$value is more efficient as compared to the competitor control charts [[23](#_ENREF_23)].

Without any loss of generality, we choose $\mu=0$ and $\sigma=1$. Suppose, because of some assignable cause, the IC process to be shifted as $\mu^{*}=\mu+\delta\sigma$. For comparison purpose, we assumed the same hyper-parameters i.e. $\left( a=10,b=4 \right)$, and the same current and future sample size as taken by Riaz *et al.* [[18](#_ENREF_18)]. Following [[52](#_ENREF_52), [18](#_ENREF_18)], we assumed $c=4$ in LLF. The $ARL$ values of the proposed chart are presented in the Table 1-3.

**[Table 1-3 and Fig 1-3 paste here]**

Some results obtained by this study are given as

- The control chart coefficient, *L* of the Bayesian Modified-EWMA chart increases by increasing the smoothing constant for all loss functions to be used in this study.
- The ${ARL}_{1}$ behavior of the proposed chart is similar under various loss functions including SELF, WBLF, PLF, and LLF. For a very small shift, the ${ARL}_{1}$ of the proposed chart decreases quickly. The ${ARL}_{1}$decreases by increasing shift for all loss functions used here (cf. Fig 1-3).
- For the fixed value of $\lambda,$the performance of the proposed chart increases by increasing the sample size under SELF, WBLF, PLF, and LLF. This implies that an OC signal is noticed early for a large sample size as compared to a small sample size (cf. Fig 3).
- The ${ARL}_{1}$ of the proposed chart decreases by decreasing the smoothing constant and increasing the sample size for all loss functions. So, the proposed chart performs better for the small smoothing constant and for the large sample size (cf. Fig 1-3).
- The proposed chart under SELF and WBLF performs better than the proposed chart under LLF and PLF for different choices of smoothing constant and sample size (cf. Fig­ 2).

**4.1: Comparison of Proposed with Existing chart**

Here, we present a comparative study of the proposed Bayesian Modified-EWMA chart with some existing control charts present in the literature.

**4.1.1: Proposed chart vs. existing Bayesian EWMA chart**

Here, we compared the proposed Bayesian Modified-EWMA chart with the existing Bayesian EWMA chart suggested by [[18](#_ENREF_18)], which can be obtained by setting $\psi=0$ in the proposed Bayesian Modified-EWMA chart. The results of the existing Bayesian EWMA chart for ${ARL}_{0}=370$ are presented in Table 4. It is noted that the proposed Bayesian Modified-EWMA chart over-performs to the existing Bayesian EWMA chart in terms of early shift detection ability. For example, for $\delta\mu^{*}=0.025$, $\lambda=0.15$, $n=5$ and ${ARL}_{0}=370$, the ${ARL}_{1}$ is 309.75 for the proposed chart and 336.9254 for the existing Bayesian EWMA chart under SELF. Similarly, ${ARL}_{1}$ is 319.869 for the proposed chart under PLF, whereas ${ARL}_{1}$ is 346.6004 for the existing Bayesian EWMA chart under PLF. Similarly, other entries of Table 1 and Table 4 can be compared, (cf. Fig 4).

**4.1.2: Proposed chart vs. existing classical EWMA chart**

Here, we compared the proposed Bayesian Modified-EWMA chart with the existing classical EWMA chart proposed by Roberts [[7](#_ENREF_7)]. The ${ARL}_{1}$ of classical EWMA chart are presented in Table 5 for ${ARL}_{0}=370$, $\lambda=0.05$ and $n=5$. The comparison reveals that the proposed Bayesian Modified-EWMA chart performs better than the existing classical EWMA chart for the monitoring of the process location. For example, if $\delta\mu^{*}=$0.05, 0.10, 0.25, the ${ARL}_{1}$ values of the classical EWMA chart are 271.101, 189.608, 62.976, and the ${ARL}_{1}$ values of the proposed chart under SELF are 158.03, 66.21, 17.05, respectively (Table 1 vs. Table 5). In addition, the dominance of the proposed chart is presented in Fig 5.

**4.1.3: Proposed chart vs. existing Modified-EWMA chart**

The Modified-EWMA chart is proposed by Khan *et al.* [[45](#_ENREF_45)] and some results of this control chart are reported in Table 5. The proposed chart has better ${ARL}_{1}$ performance for the monitoring of small to moderate shift, however for large shifts the existing Modified-EWMA chart has better ${ARL}_{1}$ performance. For example, if $n=5, \lambda=0.30$ $\delta\mu^{*}=$0.025, 0.05, 0.1, the ${ARL}_{1}$ values of existing Modified-EWMA chart are 347.06, 259.21, 126.83, and the ${ARL}_{1}$ values of the proposed chart under SELF are 310.58, 240.67, 113.42, respectively (Table 1 vs. Table 5). In addition, the better performance of the proposed chart $\delta$ the Modified-EWMA chart can be visualized in Fig 6.

**[Table 4-5 and Fig 4-6 paste here]**

**4.2: Real-life Application 1**

Reaming is the machining process which is used a multi-edged fluted cutting to smooth or accurate size adjusting specified instrument in the existing hole in the machine. It is used to cut the hole within +0.0005 inch of tool size and provide finishing to 32 micro inches [[53](#_ENREF_53)]. According to [[53](#_ENREF_53)], the screw threads are used for various purposes and have many applications in the machine tool industry. To hold and fasten parts together, screws, bolts, and nuts are used in the mechanical industry especially in engines of different vehicles and airplanes. According to Decheffre *et al.* [[54](#_ENREF_54)], the quality of the reaming process is much affected by the alignment of the machine parts, reamer geometry, cutting condition, and lubrication. Any variation in alignment may detract from the accuracy of the hole. All these reamers provide smooth and accurate holes in metals to maintain high quality. The quality of the reaming process is evaluated through surface roughness, diameter, and roundness etc. [[54](#_ENREF_54)].

Zhang [[55](#_ENREF_55)] and Riaz and Ali [[41](#_ENREF_41)] used a data set in the form of the summary statistics (mean and variance) for 20 samples of *n*=5, which is about the surface roughness of reamed holes in a particular metal part. These summary statistics are presented in Table 6. The data set is well fitted by a normal distribution with mean and variance are 32.1 and 21.833, respectively [[41](#_ENREF_41)]. Following Riaz and Ali [[41](#_ENREF_41)], we assume prior distribution as $\mu\sim N(30,20)$ and posterior distribution as $\mu|\boldsymbol{y\sim}Normal\left( 31.72367, 3.584086 \right)$. Using these information and real-life data given in Table 6, the graphical display of the proposed and existing charts is presented in Fig 7. Fig 7 shows the comparison between Bayesian EWMA and proposed Bayesian Modified-EWMA control chart under different loss functions with $\lambda=0.30$. In Fig 7 and 8, LCL (existing) and UCL (existing) refer to control limits of the existing Bayesian EWMA chart, whereas, the LCL (proposed) and UCL (proposed) refer to control limits of the proposed Bayesian Modified-EWMA chart. Similarly, $Z_{t}$ (existing) and $Z_{t}$ (proposed) are the plotting statistics for the existing Bayesian EWMA chart and the proposed Bayesian Modified-EWMA chart, respectively. From Fig7, the proposed Bayesian Modified-EWMA chart performs better than the existing Bayesian EWMA chart in terms of early shift detection. For example, the proposed chart detects first OC signal at sample number 3 under PLF whereas the existing chart detects first OC signal at sample number 8. Also, the proposed chart detects more OC signals in the case of SELF, PLF, and LLF as compared to the existing control chart (see Fig7).

**4.2: Real-life Application 2**

Manufacturing companies of golf balls spend a lot of money on the research to improve the performance of golf balls for maximum flight distance and used the latest techniques to improve the performance of these balls. The performance of the golf balls depends upon the conditions, material (silicone, titanium and several kinds of urethane), engineer expertise and the equipment to be used for the manufacturing of balls (http://www.all-science-fair-projects.com/print_project_1221_57). For more detailed information on golf ball see [[56](#_ENREF_56)].

Savran *et al.*[[57](#_ENREF_57)] designed the technical report entitled “The Golf Ball Detector” at the Department of Mechanical, Industrial and Manufacturing Engineering, Northeastern University. They stated that the United States Golf Association (USGA) performs different tests to check the golf ball and club design and its performance. The USGA uses the robotic arm called “Iron Byron” and the golf club to check the performance of a golf ball in terms of swing and movement of the golf ball. The USGA also detects the strength of thermoplastic, covering, inertia and the compressing ability (named coefficient of restitution) of the ball. A simple bonce of a golf ball test is performed for the coefficient of restitution. A bounce test is also performed to check the effect of tag on the behavior of the prototype.

In this subsection, we presented the implementation and performance of the proposed Bayesian Modified-EWMA control chart under various loss functions. For this purpose, we considered a real-life data set from the book entitled, “Introduction to Bayesian Statistics (second edition)”, by William M. Bolstad page: 236, [[58](#_ENREF_58)]. The data explain the golf ball first bonce when the 10 new golf balls dropped down from the height of 1 meter. The bounces of these 10 balls are given in Table 7.

Assuming the height (in cm) a golf ball bounces when dropped from the height of one meter is $Normal\left( \mu,\sigma^{2} \right)$, where $\sigma=12$. Using a prior distribution for $\mu$ is $Normal\left( 75,{10}^{2} \right)$. We set the $ARL_{0}$ as 370 for a valid comparison of existing Bayesian EWMA with the proposed Bayesian Modified-EWMA chart. For this purpose, we considered the real-life data and the prior distribution of mean $\mu$ of the golf ball bounce to obtained posterior predictive distribution for the monitoring of golf ball bounce. Fig 8 shows the existing Bayesian EWMA and the proposed Bayesian Modified-EWMA control chart under various loss functions at $\lambda$=0.15. The EWMA statistic and the Modified-EWMA statistic are plotted to their corresponding control limits for each loss function. From Fig 8, the proposed Bayesian Modified-EWMA chart performs better than the existing Bayesian EWMA chart for the monitoring of a quick shift in the process. For example, the proposed chart detects an OC signal at sample number 4 whereas the existing Bayesian EWMA chart does not show any OC signal under SELF, PLF, and LLF. Hence, the proposed Bayesian Modified-EWMA chart has a dominant performance than the existing Bayesian EWMA chart.

**[Table 6 and Fig 7-8 paste here]**

**5: Summary and Conclusions**

This article presents a new Bayesian Modified-EWMA chart based on posterior predictive distribution under various loss functions for efficient process monitoring of the location parameter. The ARL is used as a tool to measure the performance of the proposed chart. A simulation study is conducted to judge the behavior of ARL. We used conjugate prior and hyper-parameters from the previous study. The performance of the proposed chart under SELF and WBLF is better than the proposed chart under LLF and PLF. Similarly, the proposed chart performed better for smaller smoothing constant and for larger sample sizes under all these loss functions to be used here. It is observed that the proposed Bayesian Modified-EWMA chart under different loss functions detects earlier shifts that occur in the process location. A detailed comparison of the proposed chart with the existing charts is also provided. The study is also supported by two real-life applications.

This study may be extended to the non-normal distributions and authors are already working on these sides. The multivariate structure of the proposed chart is worthwhile for practical purpose and need to be explored.

**References**

1. Antony J, Balbontin A, Taner T. Key ingredients for the effective implementation of statistical process control.

Work Study. 2000;49(6):242-7.

2. Economic control of quality manufactured product [Internet]. D. Van Nostrand(reprinted by the American Society for Quality Control in 1980, Milwaukee, WI). 1931.

3. Stoumbos ZG, Reynolds MRJ, Ryan TP, Woodall WH. The state of statistical process control as we proceed into the 21st century. Journal of the American Statistical Association. 2002;95:992-8.

4. Hawkins DM, Olwell DH. Cumulative Sum Charts and Charting for Quality Improvement. New York: Springer; 1998.

5. Reynolds MR, Stoumbos ZG. Comparisons of some exponentially weighted moving average control charts for monitoring the process mean and variance. Technometrics. 2006;48(4):550–67.

6. Montgomery DC. Introduction to Statistical Quality Control. 6th ed. New York: John Willey & Sons; 2009.

7. Roberts SW. Control Chart Tests Based on Geometric Moving Averages. Technometrics. 1959;1(3):239-50.

8. Crowder SV, Hamilton MD. An EWMA for Monitoring a Process Standard Deviation. Journal of Quality Technology. 1992;24(1):12-21. doi: 10.1080/00224065.1992.11979369.

9. Gan FF. Joint monitoring of process mean and variance using exponentially weighted moving average control charts. Technometrics. 1995;37(4):446-53.

10. Acosta-Mejia CA, Pignatiello JJ, Venkateshwara RB. A comparison of control charting procedures for monitoring process dispersion. IIE Transactions. 1999;31(6):569-79.

11. Saghir A, Lin Z, Abbasi SA, Ahmad S. The Use of Probability Limits of COM–Poisson Charts and their Applications. Quality and Reliability Engineering International. 2013;29(5):759–70.

12. Abbas N, Riaz M, Does RJMM. An EWMA-Type Control Chart for Monitoring the Process Mean Using Auxiliary Information. Communications in Statistics - Theory and Methods. 2014;43(16):3485-98.

13. Lee SH, Park JH, Jun C. An exponentially weighted moving average chart controlling false discovery rate. J Stat Comput Simul 2014;48(8):1830-40.

14. Chen G, Cheng SW, Xie H. A New EWMA Control Chart for Monitoring Both Location and Dispersion. Quality Technology & Quantitative Management. 2016;1(2):217-31.

15. Osei-Aning R, Abbasi SA, Riaz M. Mixed EWMA-CUSUM and mixed CUSUM-EWMA modified control charts for monitoring first order autoregressive processes. Quality Technology & Quantitative Management. 2017;14(4):429-53.

16. Lu SL, Huang CJ. Statistically constrained economic design of maximum double EWMA control charts based on loss functions Quality Technology & Quantitative Management. 2017;14(3):280-95.

17. Aslam M, Saghir A, Ahmad L, Jun C, Hussain J. A control chart for COM-Poisson distribution using a modified EWMA statistic. Journal of Statistical Computation and Simulation. 2017;87(18):3491-502.

18. Riaz S, Riaz M, Hussain Z, Abbas T. Monitoring the performance of Bayesian EWMA control chart using loss functions. Computers & Industrial Engineering. 2017;112:426-36.

19. Ajadi JO, Riaz M. Mixed multivariate EWMA-CUSUM control charts for an improved process monitoring. Communications in Statistics - Theory and Methods. 2017;46(14):6980-93.

20. Quinino RDC, Ho LL, Trindade ALG. Estimation in X-bar control charts: effects and corrections. Int J Adv Manuf Technol. 2014;72(1-4):101-6.

21. Chen F, Chen S. Injury severities of truck drivers in single- and multi-vehicle accidents on rural highways. Accident Analysis and Prevention. 2011;43:1677-88.

22. Chen F, Song M, Ma X. Investigation on the Injury Severity of Drivers in Rear-End Collisions Between Cars Using a Random Parameters Bivariate Ordered Probit Model. International journal of envirnomental research and public health. 2019;16:1-12.

23. Riaz S, Riaz M, Nazeer A, Hussain Z. On Bayesian EWMA control charts under different loss functions. Qual Reliab Engng Int. 2017;33(8):2653-65.

24. Zeng Q, Wen H, Huang H, Abdel-Aty M. A Bayesian spatial random parameters Tobit model for analyzingcrash rates on roadway segments. Accident Analysis and Prevention 100 (2017) 37–43. 2017;100:37-43.

25. Zeng Q, Guo Q, Wong SC, Wen H, Huang H, Pei X. Jointly modeling area-level crash rates by severity: A Bayesian multivariate random-parameters spatio-temporal Tobit regression. Transportmetrica A: Transport Science. 2019;15(2):1867-84.

26. Zeng Q, Wen H, Huang H, Pei X, Wong SC. Incorporating temporal correlation into a multivariate random parameters Tobit model for modeling crash rate by injury severity. Transportmetrica A: Transport Science. 2016;14(3):177-91.

27. Zeng Q, Gub W, Zhang X, Wen H, Lee J, Hao W. Analyzing freeway crash severity using a Bayesian spatial generalized ordered logit model with conditional autoregressive priors. Accident Analysis and Prevention. 2019;127:87-95.

28. Girshick MA, Rubin H. A Bayes approach to a quality control model. The Annals of Mathematical Statistics. 1952:114-25.

29. Biswas S. Statistics of Quality Control. London: New International Pvt. Ltd.; 1975.

30. Montgomery DC. Introduction to Statistical Quality Control New York: John Wiley and Sons; 1991.

31. Brush GG. A comparison of classical and Bays producer’s risk. Technometrics. 1986;28(1):69–72.

32. Sharma KK, Bhuttani RK. A comparison of classical and Bayes risks when quality varies randomly. Microelectron Reliability. 1992;32(4):493–5.

33. Menzefricke U. Control charts for the generalized variance based on its predictive distribution Communications in Statistics-Theory and Methods. 2007;36(5):1031–8.

34. Menzefricke U. On the evaluation of control chart limits based on predictive distributions Communications in Statistics-Theory and Methods. Communications in Statistics-Theory and Methods. 2002;31(8):1423-40.

35. Saghir A. Phase-I Design Scheme for -chart Based on Posterior Distribution. Communications in Statistics - Theory and Methods. 2015;44(3):644-55.

36. Menzefricke U. Combined exponentially weighted moving average charts for the mean and variance based on the predictive distribution. Communications in Statistics-Theory and Methods. 2013a;42(22):4003–16.

37. Menzefricke U. Control charts for the mean and variance based on changepoint methodology. Communications in Statistics-Theory and Methods. 2013b;42(6):988–1007.

38. Ali S, Riaz M. On Designing a New Bayesian Dispersion Chart for Process Monitoring. Arabian Journal for Science and Engineering. 2019. doi: https://doi.org/10.1007/s13369-019-04036-w.

39. Wu Z, Tian Y. Weighted-loss-function cusum chart for monitoring mean and variance of a production process. International Journal of Production Research. 2005;43(14):3027–44.

40. Serel DA. Economic design of EWMA control charts based on loss function. Mathematical and Computer Modelling. 2009;49(3):745–59.

41. Riaz M, Ali S. On process monitoring using location control charts under different loss functions. Transactions of the Institute of Measurement and Control. 2015;38(9):1-13.

42. Tsui KL, Woodall WH. Multivariate control charts based on loss functions. Sequential Analysis. 1993;12(1):79–92.

43. Ben-Daya M, Duffuaa SO. Integration of Taguchi’s loss function approach in the economic design of x-chart. International Journal of Quality & Reliability Management. 2003;20(5): 607–19.

44. Lavanya A, Alexander TL. Bayesian estimation of the failure rate using extension of Jeffreys pior information with three loss functions International Journal of Science and Research. 2016;5(9):736–42.

45. Khan N, Aslam M, Jun C. Design of a Control Chart Using a Modified EWMA Statistic. Qual Reliab Engng Int 2017;33(5):1095-104.

46. Anwar SM, Aslam M, Ahmad S, Riaz M. A Modified-mxEWMA location Chart for the Improved Process Monitoring Using Auxiliary Information and Its Application in Wood Industry. Quality Technology & Quantitative Management. 2019. doi: https://doi.org/10.1080/16843703.2019.1696011.

47. Saghir A, Aslam M, Faraz A, Ahmad L, Heuchenne C. Monitoring process variation using modified EWMA. Qual Reliab Engng Int. 2019. doi: https://doi.org/10.1002/qre.2576.

48. Sameniego FJ. A Comparison of the Bayesian and Frequentist Approaches to Estimation. New York, London: Springer; 2010.

49. Varian HR. A bayesian approach to real estate assessment. Studies in Bayesian econometrics and statistics in honor of Leonard. Journal of Savage. 1975:195–208.

50. Rodrigues J. Bayesian estimation of a normal mean parameter using the linex loss function and robustness considerations. Test. 1994;3(2):237–46.

51. Norstrøm JG. The use of precautionary loss functions in risk analysis. IEEE Transactions on Reliability. 1996;45(3): 400–3.

52. Zeller A. Bayesian estimation and prediction using asymmetric loss function Journal of the American Statistical Association. 1986;81:446-51.

53. Schneider G. Cutting Tool Applications. Tooling and Productions. 2010.

54. De Chiffre L, Tosello G, Piska M, Müller P, editors. Investigation on Capability of Reaming Process using Minimal Quantity Lubrication. Proceedings of The Swedish Production Symposium; 2008: Royal Institute of Technology Stockholm, Sweden.

55. Zhang G. Improved R and S control charts for monitoring the process variance. Journal of Applied Statistics. 2014;41(6):1260-73.

56. Tanaka K, Sato F, Oodaira H, Teranishi H, Ujihashi S. Construction of the finite-element models of golf balls and simulations of their collisions. Journal of Materials: Design & Applications 2006;Part L(.):13-22.

57. Savran A, Matlo M, Abdul-Ghaffar K. The Golf Ball Detector 2002 [May 29, 2002]. 1-76]. Available from: https://repository.library.northeastern.edu/downloads/neu:377323?datastream_id=content.

58. Bolstad WM. Introduction to Bayesian statistics. London: John Wiley & Sons; 2007.

Table 1. ARL comparison for *n*=5 under various loss function for ${ARL}_{0}=370$

|  |  |  | Shift | | | | | | | | | |
| --- | --- | --- | --- | --- | --- | --- | --- | --- | --- | --- | --- | --- |
| $\lambda$ |  | L | 0 | 0.025 | 0.05 | 0.075 | 0.1 | 0.25 | 0.5 | 0.75 | 1 | 2 |
| 0.05 | SELF | 1.636 | 367.69 | 277.17 | 158.03 | 97.75 | 66.21 | 17.05 | 5.35 | 2.51 | 1.47 | 1.00 |
|  | LLF | 1.637 | 372.32 | 290.11 | 163.94 | 102.84 | 66.78 | 18.61 | 5.36 | 2.50 | 1.47 | 1.00 |
|  | WBLF | 1.635 | 370.08 | 280.13 | 169.87 | 98.54 | 70.93 | 18.06 | 5.58 | 2.58 | 1.47 | 1.00 |
|  | PLF | 1.64 | 367.34 | 291.64 | 164.90 | 103.73 | 71.23 | 18.96 | 5.40 | 2.53 | 1.55 | 1.00 |
| 0.15 | SELF | 1.92 | 368.04 | 309.75 | 215.22 | 131.93 | 85.77 | 20.14 | 6.07 | 2.88 | 1.71 | 1.00 |
|  | LLF | 1.922 | 372.49 | 312.04 | 215.48 | 144.24 | 86.73 | 20.18 | 6.02 | 2.89 | 1.78 | 1.01 |
|  | WBLF | 1.92 | 369.68 | 315.15 | 227.31 | 139.41 | 90.44 | 20.21 | 6.04 | 2.90 | 1.70 | 1.01 |
|  | PLF | 1.9154 | 368.34 | 319.87 | 220.10 | 145.58 | 91.88 | 20.98 | 6.19 | 2.93 | 1.75 | 1.01 |
| 0.3 | SELF | 2.074 | 371.02 | 310.58 | 240.67 | 160.59 | 113.42 | 22.31 | 6.14 | 2.89 | 1.74 | 1.01 |
|  | LLF | 2.079 | 369.73 | 322.57 | 242.68 | 161.85 | 117.75 | 23.26 | 6.25 | 2.91 | 1.77 | 1.01 |
|  | WBLF | 2.073 | 368.52 | 326.91 | 248.42 | 171.69 | 113.33 | 22.66 | 6.16 | 2.91 | 1.77 | 1.01 |
|  | PLF | 2.068 | 372.19 | 337.01 | 249.33 | 172.44 | 122.84 | 24.87 | 6.28 | 2.96 | 1.76 | 1.01 |
| 0.7 | SELF | 2.317 | 368.60 | 333.80 | 284.22 | 225.39 | 172.76 | 37.31 | 7.37 | 2.63 | 1.66 | 1.00 |
|  | LLF | 2.31 | 368.92 | 345.26 | 296.41 | 225.72 | 173.30 | 37.39 | 6.71 | 2.80 | 1.69 | 1.00 |
|  | WBLF | 2.313 | 367.91 | 341.36 | 298.83 | 234.49 | 181.71 | 37.94 | 7.35 | 2.73 | 1.67 | 1.00 |
|  | PLF | 2.311 | 369.61 | 349.06 | 304.95 | 257.43 | 176.19 | 39.10 | 7.76 | 2.80 | 1.70 | 1.01 |

Table 2. ARL comparison for *n*=10 under various loss function for ${ARL}_{0}=370$

|  |  |  | shift | | | | | | | | | |
| --- | --- | --- | --- | --- | --- | --- | --- | --- | --- | --- | --- | --- |
| $\lambda$ |  | L | 0 | 0.025 | 0.05 | 0.075 | 0.1 | 0.25 | 0.5 | 0.75 | 1 | 2 |
| 0.05 | SELF | 1.644 | 368.30 | 219.22 | 105.13 | 60.09 | 39.66 | 9.14 | 2.82 | 1.36 | 1.07 | 1.00 |
|  | LLF | 1.63 | 371.50 | 222.49 | 107.33 | 61.66 | 39.82 | 9.96 | 2.80 | 1.37 | 1.08 | 1.00 |
|  | WBLF | 1.634 | 372.28 | 224.97 | 109.03 | 63.54 | 40.68 | 10.23 | 2.86 | 1.41 | 1.07 | 1.00 |
|  | PLF | 1.632 | 369.65 | 225.62 | 109.90 | 66.62 | 41.02 | 10.24 | 2.86 | 1.41 | 1.08 | 1.00 |
| 0.15 | SELF | 1.91 | 370.50 | 225.79 | 137.48 | 80.55 | 51.28 | 10.41 | 3.14 | 1.52 | 1.10 | 1.00 |
|  | LLF | 1.909 | 372.47 | 253.83 | 141.07 | 80.79 | 52.03 | 10.84 | 3.17 | 1.53 | 1.12 | 1.00 |
|  | WBLF | 1.918 | 368.43 | 270.12 | 147.78 | 82.85 | 51.29 | 10.71 | 3.25 | 1.51 | 1.10 | 1.00 |
|  | PLF | 1.913 | 369.90 | 273.25 | 146.23 | 85.17 | 51.94 | 11.10 | 3.27 | 1.54 | 1.11 | 1.00 |
| 0.3 | SELF | 2.067 | 368.09 | 235.18 | 113.97 | 54.04 | 33.75 | 5.93 | 1.72 | 1.08 | 1.00 | 1.00 |
|  | LLF | 2.075 | 367.77 | 244.10 | 120.79 | 55.68 | 35.88 | 5.99 | 1.74 | 1.10 | 1.00 | 1.00 |
|  | WBLF | 2.069 | 368.21 | 244.79 | 114.18 | 56.97 | 35.97 | 5.97 | 1.80 | 1.10 | 1.01 | 1.00 |
|  | PLF | 2.066 | 367.59 | 246.35 | 126.83 | 61.15 | 38.76 | 6.05 | 1.83 | 1.09 | 1.00 | 1.00 |
| 0.7 | SELF | 2.307 | 369.02 | 263.66 | 164.65 | 96.33 | 59.47 | 6.81 | 1.67 | 1.07 | 1.00 | 1.00 |
|  | LLF | 2.308 | 372.74 | 288.26 | 167.29 | 96.71 | 59.99 | 7.19 | 1.76 | 1.07 | 1.01 | 1.00 |
|  | WBLF | 2.312 | 371.54 | 274.11 | 180.29 | 102.20 | 59.54 | 6.95 | 1.69 | 1.08 | 1.00 | 1.00 |
|  | PLF | 2.3085 | 372.77 | 298.83 | 183.87 | 103.12 | 60.11 | 7.32 | 1.77 | 1.08 | 1.01 | 1.00 |

Table 3. ARL comparison for *n*=20 under various loss function for ${ARL}_{0}=370$

|  |  |  | shift | | | | | | | | | |
| --- | --- | --- | --- | --- | --- | --- | --- | --- | --- | --- | --- | --- |
| $\lambda$ |  | L | 0 | 0.025 | 0.05 | 0.075 | 0.1 | 0.25 | 0.5 | 0.75 | 1 | 2 |
| 0.05 | SELF | 1.63 | 369.13 | 160.16 | 65.77 | 37.98 | 24.87 | 5.26 | 1.48 | 1.04 | 1.00 | 1 |
|  | LLF | 1.635 | 368.87 | 174.86 | 69.93 | 39.65 | 25.44 | 5.56 | 1.49 | 1.05 | 1.00 | 1 |
|  | WBLF | 1.6282 | 371.67 | 161.31 | 68.56 | 40.23 | 25.97 | 5.56 | 1.50 | 1.04 | 1.00 | 1 |
|  | PLF | 1.6318 | 368.34 | 177.27 | 69.33 | 41.88 | 26.54 | 5.75 | 1.49 | 1.05 | 1.00 | 1 |
| 0.15 | SELF | 1.907 | 368.49 | 201.61 | 86.67 | 43.17 | 26.91 | 5.44 | 1.64 | 1.07 | 1.01 | 1 |
|  | LLF | 1.905 | 368.56 | 206.97 | 88.58 | 45.41 | 27.90 | 5.94 | 1.66 | 1.07 | 1.00 | 1 |
|  | WBLF | 1.914 | 368.19 | 209.38 | 90.28 | 45.16 | 28.80 | 6.14 | 1.65 | 1.09 | 1.00 | 1 |
|  | PLF | 1.909 | 371.46 | 210.57 | 91.45 | 46.63 | 29.33 | 6.29 | 1.71 | 1.09 | 1.00 | 1 |
| 0.3 | SELF | 2.0672 | 368.09 | 235.18 | 113.97 | 54.04 | 33.75 | 5.93 | 1.72 | 1.08 | 1.00 | 1 |
|  | LLF | 2.075 | 367.77 | 244.10 | 120.79 | 55.68 | 35.88 | 5.99 | 1.74 | 1.10 | 1.00 | 1 |
|  | WBLF | 2.069 | 368.21 | 244.79 | 114.18 | 56.97 | 35.97 | 5.97 | 1.80 | 1.10 | 1.01 | 1 |
|  | PLF | 2.0659 | 367.59 | 246.35 | 126.83 | 61.15 | 38.76 | 6.05 | 1.83 | 1.09 | 1.00 | 1 |
| 0.7 | SELF | 2.307 | 369.02 | 263.66 | 164.65 | 96.33 | 59.47 | 6.81 | 1.67 | 1.07 | 1.00 | 1 |
|  | LLF | 2.308 | 372.74 | 288.26 | 167.29 | 96.71 | 59.99 | 7.19 | 1.76 | 1.07 | 1.01 | 1 |
|  | WBLF | 2.312 | 371.54 | 274.11 | 180.29 | 102.20 | 59.54 | 6.95 | 1.69 | 1.08 | 1.00 | 1 |
|  | PLF | 2.3085 | 372.77 | 298.83 | 183.87 | 103.12 | 60.11 | 7.32 | 1.77 | 1.08 | 1.01 | 1 |

Table 4. ARLs of existing Bayesian EWMA chart for *n*=5 and $\lambda=0.15$ under various loss function for ${ARL}_{0}=370$

|  |  |  |  |  |  |  | Shifts |  |  |  |  |  |
| --- | --- | --- | --- | --- | --- | --- | --- | --- | --- | --- | --- | --- |
|  | L | 0 | 0.025 | 0.05 | 0.075 | 0.1 | 0.25 | 0.5 | 0.75 | 1 | 1.25 | 1.5 |
| SELF | 2.136 | 373.35 | 336.93 | 273.62 | 203.18 | 144.25 | 30.58 | 9.30 | 5.205 | 3.701 | 2.894 | 2.409 |
| PLF | 1.7 | 368.31 | 346.6 | 316.31 | 284.29 | 253.92 | 107.74 | 28.33 | 11.829 | 6.692 | 4.529 | 3.395 |
| LLF | 2.35 | 367.40 | 341.39 | 281.29 | 222.42 | 163.18 | 36.85 | 10.64 | 5.935 | 4.140 | 3.191 | 2.647 |

Table 5. ARLs of existing classical EWMA and Modified-EWMA chart for *n*=5 and ${ARL}_{0}=370$

|  |  |  |  |  |  |  | Shift |  |  |  |  |  |
| --- | --- | --- | --- | --- | --- | --- | --- | --- | --- | --- | --- | --- |
| $\lambda$ |  | L | 0 | 0.025 | 0.05 | 0.1 | 0.25 | 0.5 | 0.75 | 1 | 1.5 | 2 |
| 0.05 | EWMA | 2.5350 | 369.00 | - | 271.104 | 189.608 | 62.976 | 20.086 | 10.238 | 6.344 | 3.276 | 2.078 |
| 0.3 | Modified- EWMA | 2.99967 | 370.00 | 347.06 | 259.21 | 126.83 | - | 2.2 | - | 1 | - | - |

Table 6. Real life data-1 (Summary statistics of 20 samples each containing 5 surface roughness measurement on reamed hole (in))

| Sample no. | 1 | 2 | 3 | 4 | 5 | 6 | 7 | 8 | 9 | 10 |
| --- | --- | --- | --- | --- | --- | --- | --- | --- | --- | --- |
| Mean | 34.6 | 46.8 | 32.6 | 42.6 | 26.6 | 29.6 | 33.6 | 28.2 | 25.8 | 32.6 |
| Variance | 11.56 | 77.44 | 21.16 | 7.29 | 5.76 | 0.81 | 36 | 6.25 | 10.24 | 56.25 |
| Sample no. | 11 | 12 | 13 | 14 | 15 | 16 | 17 | 18 | 19 | 20 |
| Mean | 34 | 34.8 | 36.2 | 27.4 | 27.2 | 32.8 | 31 | 33.8 | 30.8 | 21 |
| Variance | 82.81 | 3.61 | 1.69 | 92.16 | 1.69 | 4.84 | 6.25 | 7.29 | 2.56 | 1 |

Table 7. Real-life data set-2 (Golf ball first bounces of 10 balls from the distance of 1-meter measurement in cm)

| Ball # | 1 | 2 | 3 | 4 | 5 | 6 | 7 | 8 | 9 | 10 |
| --- | --- | --- | --- | --- | --- | --- | --- | --- | --- | --- |
| Bounce(cm) | 79.9 | 80.0 | 78.9 | 78.5 | 75.6 | 80.5 | 82.5 | 80.1 | 81.6 | 76.7 |
