# Supplementary material for: An improved Bayesian Modified-EWMA location chart and its applications in mechanical and sport industry
Source: PLoS One. 2020 Feb 26;15(2):e0229422. doi: 10.1371/journal.pone.0229422 (PMC7043768; doi:10.1371/journal.pone.0229422)
Supplement: S2 File — (DOCX) [file pone.0229422.s002.docx]

|  |  |  | SELF |  |  |  |  |  | LLF |  |  |
| --- | --- | --- | --- | --- | --- | --- | --- | --- | --- | --- | --- |
| $\lambda$\Shift | 0.025 | 0.05 | 0.075 | 0.1 | 0.25 |  | 0.025 | 0.05 | 0.075 | 0.1 | 0.25 |
| 0.05 | 277.17 | 158.03 | 97.75 | 66.21 | 17.05 |  | 290.11 | 163.94 | 102.84 | 66.78 | 18.61 |
| 0.15 | 309.75 | 215.22 | 131.93 | 85.77 | 20.14 |  | 312.04 | 215.48 | 144.24 | 86.73 | 20.18 |
| 0.30 | 310.58 | 240.67 | 160.59 | 113.42 | 22.31 |  | 322.57 | 242.68 | 161.85 | 117.75 | 23.26 |
| 0.70 | 333.80 | 284.22 | 225.39 | 172.76 | 37.31 |  | 345.26 | 296.41 | 225.72 | 173.30 | 37.39 |

Figure 1 data taken from table 1

Figure 2 data taken from table 3

| $\lambda$ |  |  | 0.30 |  |  |  |  |  | 0.70 |  |  |
| --- | --- | --- | --- | --- | --- | --- | --- | --- | --- | --- | --- |
| Shift | 0.025 | 0.05 | 0.075 | 0.1 | 0.25 |  | 0.025 | 0.05 | 0.075 | 0.1 | 0.25 |
| SELF | 235.18 | 113.97 | 54.04 | 33.75 | 5.93 |  | 263.66 | 164.65 | 96.33 | 59.47 | 6.81 |
| LLF | 244.10 | 120.79 | 55.68 | 35.88 | 5.99 |  | 288.26 | 167.29 | 96.71 | 59.99 | 7.19 |
| WBLF | 244.79 | 114.18 | 56.97 | 35.97 | 5.97 |  | 274.11 | 180.29 | 102.20 | 59.54 | 6.95 |
| PLF | 246.35 | 126.83 | 61.15 | 38.76 | 6.05 |  | 298.83 | 183.87 | 103.12 | 60.11 | 7.32 |

Figure 3 data taken from table 1-3 at $\lambda=0.05$

| Shift | 0.025 | 0.05 | 0.075 | 0.1 | 0.25 | 0.5 | 0.75 | 1 |
| --- | --- | --- | --- | --- | --- | --- | --- | --- |
| $n$ | SELF | | | | | | | |
| 5 | 277.17 | 158.03 | 97.75 | 66.21 | 17.05 | 5.35 | 2.51 | 1.47 |
| 10 | 219.22 | 105.13 | 60.09 | 39.66 | 9.14 | 2.82 | 1.36 | 1.07 |
| 20 | 160.16 | 65.77 | 37.98 | 24.87 | 5.26 | 1.48 | 1.04 | 1.00 |
|  | LLF | | | | | | | |
| 5 | 290.11 | 163.94 | 102.84 | 66.78 | 18.61 | 5.36 | 2.50 | 1.47 |
| 10 | 222.49 | 107.33 | 61.66 | 39.82 | 9.96 | 2.80 | 1.37 | 1.08 |
| 20 | 174.86 | 69.93 | 39.65 | 25.44 | 5.56 | 1.49 | 1.05 | 1.00 |

Figure 4 data taken from table 1 and 4 at $\lambda=0.15$ and n=5

| Shift | 0.025 | 0.05 | 0.075 | 0.1 | 0.25 | 0.5 | 0.75 | 1 |
| --- | --- | --- | --- | --- | --- | --- | --- | --- |
|  | SELF | | | | | | | |
| Proposed | 309.75 | 215.22 | 131.93 | 85.77 | 20.14 | 6.07 | 2.88 | 1.71 |
| Bayesian EWMA | 336.93 | 273.62 | 203.18 | 144.25 | 30.58 | 9.30 | 5.205 | 3.701 |
|  | LLF | | | | | | | |
| Proposed | 312.04 | 215.48 | 144.24 | 86.73 | 20.18 | 6.02 | 2.89 | 1.78 |
| Bayesian EWMA | 341.39 | 281.29 | 222.42 | 163.18 | 36.85 | 10.64 | 5.935 | 4.140 |

Figure 5 data taken from table 1 and 5 at $\lambda=0.05$ and n=5

| Shift | 0.05 | 0.1 | 0.25 | 0.50 | 0.75 | 1 |
| --- | --- | --- | --- | --- | --- | --- |
| Proposed  (SELF) | 158.03 | 66.21 | 17.05 | 5.35 | 2.51 | 1.47 |
| Classical EWMA | 271.104 | 189.608 | 62.976 | 20.086 | 10.238 | 6.344 |

Figure 6 data taken from table 1 and 5 at $\lambda=0.30$ and n=5

| Shift | 0.025 | 0.05 | 0.1 | 0.5 | 1 |
| --- | --- | --- | --- | --- | --- |
| Proposed  (SELF) | 310.58 | 240.67 | 113.42 | 6.14 | 1.74 |
| Modified- EWMA | 347.06 | 259.21 | 126.83 | 2.2 | 1 |

Figure 7 data (bold) of existing Bayesian EWMA and proposed Bayesian modified-EWMA chart to real-life data-I under SELF

| Sample | $\bar{Y}_{t}$ | $\left. \bar{X}_{t} \right\vert\boldsymbol{y}$ | $Z_{t}$ | LCL | UCL | $Z_{t}$ | LCL | UCL |
| --- | --- | --- | --- | --- | --- | --- | --- | --- |
| No. |  |  | existing | existing | existing | Proposed | Proposed | Proposed |
| 1 | 34.60 | 32.329 | **32.032** | **31.778** | **32.205** | **31.997** | **31.805** | **32.178** |
| 2 | 46.80 | 31.444 | **31.855** | **31.731** | **32.252** | **31.964** | **31.764** | **32.219** |
| 3 | 32.60 | 31.726 | **31.817** | **31.711** | **32.272** | **31.774** | **31.746** | **32.237** |
| 4 | 42.60 | 32.328 | **31.970** | **31.701** | **32.281** | **31.880** | **31.738** | **32.245** |
| 5 | 26.60 | 31.670 | **31.880** | **31.697** | **32.286** | **31.979** | **31.734** | **32.249** |
| 6 | 29.60 | 31.863 | **31.875** | **31.695** | **32.288** | **31.846** | **31.732** | **32.251** |
| 7 | 33.60 | 31.477 | **31.755** | **31.694** | **32.289** | **31.813** | **31.731** | **32.252** |
| 8 | 28.20 | 31.850 | **31.784** | **31.693** | **32.289** | **31.728** | **31.731** | **32.252** |
| 9 | 25.80 | 31.935 | **31.829** | **31.693** | **32.290** | **31.816** | **31.730** | **32.252** |
| 10 | 32.60 | 31.772 | **31.812** | **31.693** | **32.290** | **31.836** | **31.730** | **32.252** |
| 11 | 34.00 | 31.623 | **31.755** | **31.693** | **32.290** | **31.777** | **31.730** | **32.252** |
| 12 | 34.80 | 31.900 | **31.798** | **31.693** | **32.290** | **31.757** | **31.730** | **32.252** |
| 13 | 36.20 | 31.809 | **31.802** | **31.693** | **32.290** | **31.815** | **31.730** | **32.252** |
| 14 | 27.40 | 32.139 | **31.903** | **31.693** | **32.290** | **31.853** | **31.730** | **32.252** |
| 15 | 27.20 | 32.095 | **31.961** | **31.693** | **32.290** | **31.967** | **31.730** | **32.252** |
| 16 | 32.80 | 32.135 | **32.013** | **31.693** | **32.290** | **32.007** | **31.730** | **32.252** |
| 17 | 31.00 | 32.229 | **32.078** | **31.693** | **32.290** | **32.064** | **31.730** | **32.252** |
| 18 | 33.80 | 31.529 | **31.913** | **31.693** | **32.290** | **32.018** | **31.730** | **32.252** |
| 19 | 30.80 | 32.183 | **31.994** | **31.693** | **32.290** | **31.896** | **31.730** | **32.252** |
| 20 | 21.00 | 32.048 | **32.010** | **31.693** | **32.290** | **32.031** | **31.730** | **32.252** |

Figure 7 data (bold) of existing Bayesian EWMA and proposed Bayesian modified-EWMA chart to real-life data-I under PLF

| Sample | $\bar{Y}_{t}$ | $\left. \bar{X}_{t} \right\vert\boldsymbol{y}$ | $Z_{t}$ | LCL | UCL | $Z_{t}$ | LCL | UCL |
| --- | --- | --- | --- | --- | --- | --- | --- | --- |
| No. |  |  | existing | existing | existing | Proposed | Proposed | Proposed |
| 1 | 34.60 | 32.870 | **32.436** | **32.163** | **32.592** | **32.343** | **32.191** | **32.564** |
| 2 | 46.80 | 31.580 | **32.179** | **32.116** | **32.639** | **32.373** | **32.150** | **32.605** |
| 3 | 32.60 | 31.991 | **32.123** | **32.096** | **32.659** | **32.061** | **32.132** | **32.623** |
| 4 | 42.60 | 32.869 | **32.347** | **32.086** | **32.669** | **32.215** | **32.124** | **32.631** |
| 5 | 26.60 | 31.908 | **32.215** | **32.082** | **32.673** | **32.359** | **32.120** | **32.635** |
| 6 | 29.60 | 32.190 | **32.208** | **32.080** | **32.675** | **32.165** | **32.118** | **32.637** |
| 7 | 33.60 | 31.627 | **32.033** | **32.079** | **32.677** | **32.118** | **32.117** | **32.638** |
| 8 | 28.20 | 32.171 | **32.075** | **32.078** | **32.677** | **31.993** | **32.117** | **32.638** |
| 9 | 25.80 | 32.295 | **32.141** | **32.078** | **32.677** | **32.122** | **32.117** | **32.638** |
| 10 | 32.60 | 32.057 | **32.116** | **32.078** | **32.677** | **32.151** | **32.117** | **32.638** |
| 11 | 34.00 | 31.840 | **32.033** | **32.078** | **32.677** | **32.065** | **32.117** | **32.638** |
| 12 | 34.80 | 32.244 | **32.096** | **32.078** | **32.678** | **32.036** | **32.117** | **32.638** |
| 13 | 36.20 | 32.112 | **32.101** | **32.078** | **32.678** | **32.121** | **32.117** | **32.638** |
| 14 | 27.40 | 32.592 | **32.248** | **32.078** | **32.678** | **32.176** | **32.117** | **32.638** |
| 15 | 27.20 | 32.529 | **32.333** | **32.078** | **32.678** | **32.342** | **32.117** | **32.638** |
| 16 | 32.80 | 32.587 | **32.409** | **32.078** | **32.678** | **32.400** | **32.117** | **32.638** |
| 17 | 31.00 | 32.724 | **32.503** | **32.078** | **32.678** | **32.483** | **32.117** | **32.638** |
| 18 | 33.80 | 31.704 | **32.263** | **32.078** | **32.678** | **32.416** | **32.117** | **32.638** |
| 19 | 30.80 | 32.658 | **32.382** | **32.078** | **32.678** | **32.239** | **32.117** | **32.638** |
| 20 | 21.00 | 32.460 | **32.405** | **32.078** | **32.678** | **32.435** | **32.117** | **32.638** |

Figure 7 data (bold) of existing Bayesian EWMA and proposed Bayesian modified-EWMA chart to real-life data-I under LLF

| Sample | $\bar{Y}_{t}$ | $\left. \bar{X}_{t} \right\vert\boldsymbol{y}$ | $Z_{t}$ | LCL | UCL | $Z_{t}$ | LCL | UCL |
| --- | --- | --- | --- | --- | --- | --- | --- | --- |
| No. |  |  | existing | existing | existing | Proposed | Proposed | Proposed |
| 1 | 34.60 | 32.369 | **31.936** | **31.663** | **32.091** | **31.895** | **31.690** | **32.064** |
| 2 | 46.80 | 31.079 | **31.679** | **31.615** | **32.138** | **31.844** | **31.649** | **32.105** |
| 3 | 32.60 | 31.491 | **31.622** | **31.595** | **32.159** | **31.561** | **31.631** | **32.123** |
| 4 | 42.60 | 32.368 | **31.846** | **31.586** | **32.168** | **31.715** | **31.623** | **32.131** |
| 5 | 26.60 | 31.408 | **31.715** | **31.581** | **32.173** | **31.859** | **31.619** | **32.135** |
| 6 | 29.60 | 31.690 | **31.707** | **31.579** | **32.175** | **31.665** | **31.617** | **32.137** |
| 7 | 33.60 | 31.126 | **31.533** | **31.578** | **32.176** | **31.617** | **31.616** | **32.138** |
| 8 | 28.20 | 31.670 | **31.574** | **31.577** | **32.176** | **31.493** | **31.615** | **32.139** |
| 9 | 25.80 | 31.794 | **31.640** | **31.577** | **32.177** | **31.622** | **31.615** | **32.139** |
| 10 | 32.60 | 31.556 | **31.615** | **31.577** | **32.177** | **31.651** | **31.615** | **32.139** |
| 11 | 34.00 | 31.339 | **31.532** | **31.577** | **32.177** | **31.565** | **31.615** | **32.139** |
| 12 | 34.80 | 31.744 | **31.596** | **31.577** | **32.177** | **31.535** | **31.615** | **32.139** |
| 13 | 36.20 | 31.611 | **31.600** | **31.577** | **32.177** | **31.620** | **31.615** | **32.139** |
| 14 | 27.40 | 32.092 | **31.748** | **31.577** | **32.177** | **31.676** | **31.615** | **32.139** |
| 15 | 27.20 | 32.029 | **31.832** | **31.577** | **32.177** | **31.842** | **31.615** | **32.139** |
| 16 | 32.80 | 32.087 | **31.908** | **31.577** | **32.177** | **31.900** | **31.615** | **32.139** |
| 17 | 31.00 | 32.223 | **32.003** | **31.577** | **32.177** | **31.982** | **31.615** | **32.139** |
| 18 | 33.80 | 31.203 | **31.763** | **31.577** | **32.177** | **31.916** | **31.615** | **32.139** |
| 19 | 30.80 | 32.157 | **31.881** | **31.577** | **32.177** | **31.738** | **31.615** | **32.139** |
| 20 | 21.00 | 31.960 | **31.905** | **31.577** | **32.177** | **31.934** | **31.615** | **32.139** |

Figure 8 data (bold) of existing Bayesian EWMA and proposed Bayesian modified-EWMA chart to real-life data-II under SELF

| Sample | $Y_{t}$ | $\left. \bar{X}_{t} \right\vert\boldsymbol{y}$ | $Z_{t}$ | LCL | UCL | $Z_{t}$ | LCL | UCL |
| --- | --- | --- | --- | --- | --- | --- | --- | --- |
| No. |  |  | existing | existing | existing | Proposed | Proposed | Proposed |
| 1 | 79.9 | 79.927 | **79.360** | **79.036** | **79.789** | **79.310** | **79.165** | **79.660** |
| 2 | 80 | 78.950 | **79.299** | **78.919** | **79.906** | **79.372** | **79.087** | **79.737** |
| 3 | 78.9 | 77.733 | **79.064** | **78.849** | **79.976** | **79.155** | **79.041** | **79.783** |
| 4 | 78.5 | 79.211 | **79.086** | **78.803** | **80.021** | **78.975** | **79.011** | **79.813** |
| 5 | 75.6 | 79.666 | **79.173** | **78.772** | **80.052** | **79.139** | **78.991** | **79.834** |
| 6 | 80.5 | 78.938 | **79.138** | **78.751** | **80.074** | **79.193** | **78.977** | **79.848** |
| 7 | 82.5 | 79.697 | **79.222** | **78.736** | **80.089** | **79.165** | **78.967** | **79.858** |
| 8 | 80.1 | 78.194 | **79.068** | **78.725** | **80.099** | **79.180** | **78.960** | **79.865** |
| 9 | 81.6 | 80.467 | **79.278** | **78.718** | **80.107** | **79.107** | **78.955** | **79.870** |
| 10 | 76.7 | 79.820 | **79.359** | **78.712** | **80.112** | **79.408** | **78.952** | **79.873** |

Figure 8 data (bold) of existing Bayesian EWMA and proposed Bayesian modified-EWMA chart to real-life data-II under PLF

| Sample | $Y_{t}$ | $\left. \bar{X}_{t} \right\vert\boldsymbol{y}$ | $Z_{t}$ | LCL | UCL | $Z_{t}$ | LCL | UCL |
| --- | --- | --- | --- | --- | --- | --- | --- | --- |
| No. |  |  | existing | existing | existing | Proposed | Proposed | Proposed |
| 1 | 79.9 | 79.928 | **79.362** | **79.038** | **79.790** | **79.312** | **79.166** | **79.661** |
| 2 | 80 | 78.951 | **79.300** | **78.920** | **79.907** | **79.373** | **79.089** | **79.738** |
| 3 | 78.9 | 77.735 | **79.065** | **78.850** | **79.977** | **79.157** | **79.043** | **79.784** |
| 4 | 78.5 | 79.212 | **79.087** | **78.805** | **80.022** | **78.977** | **79.013** | **79.814** |
| 5 | 75.6 | 79.668 | **79.174** | **78.774** | **80.053** | **79.140** | **78.993** | **79.834** |
| 6 | 80.5 | 78.939 | **79.139** | **78.752** | **80.075** | **79.194** | **78.979** | **79.848** |
| 7 | 82.5 | 79.698 | **79.223** | **78.737** | **80.090** | **79.166** | **78.969** | **79.858** |
| 8 | 80.1 | 78.195 | **79.069** | **78.727** | **80.100** | **79.181** | **78.962** | **79.865** |
| 9 | 81.6 | 80.469 | **79.279** | **78.719** | **80.108** | **79.108** | **78.957** | **79.870** |
| 10 | 76.7 | 79.822 | **79.360** | **78.714** | **80.113** | **79.409** | **78.953** | **79.874** |

Figure 8 data (bold) of existing Bayesian EWMA and proposed Bayesian modified-EWMA chart to real-life data-II under LLF

| Sample | $Y_{t}$ | $\left. \bar{X}_{t} \right\vert\boldsymbol{y}$ | $Z_{t}$ | LCL | UCL | $Z_{t}$ | LCL | UCL |
| --- | --- | --- | --- | --- | --- | --- | --- | --- |
| No. |  |  | existing | existing | existing | Proposed | Proposed | Proposed |
| 1 | 79.9 | 79.597 | **79.031** | **78.815** | **79.570** | **78.981** | **78.935** | **79.450** |
| 2 | 80 | 78.620 | **78.969** | **78.697** | **79.688** | **79.042** | **78.855** | **79.530** |
| 3 | 78.9 | 77.403 | **78.734** | **78.627** | **79.758** | **78.826** | **78.807** | **79.578** |
| 4 | 78.5 | 78.881 | **78.756** | **78.581** | **79.803** | **78.645** | **78.776** | **79.609** |
| 5 | 75.6 | 79.337 | **78.843** | **78.550** | **79.834** | **78.809** | **78.755** | **79.630** |
| 6 | 80.5 | 78.608 | **78.808** | **78.529** | **79.856** | **78.863** | **78.740** | **79.645** |
| 7 | 82.5 | 79.367 | **78.892** | **78.514** | **79.871** | **78.835** | **78.730** | **79.655** |
| 8 | 80.1 | 77.864 | **78.738** | **78.503** | **79.882** | **78.850** | **78.723** | **79.662** |
| 9 | 81.6 | 80.138 | **78.948** | **78.496** | **79.889** | **78.777** | **78.717** | **79.667** |
| 10 | 76.7 | 79.490 | **79.029** | **78.490** | **79.895** | **79.078** | **78.714** | **79.671** |
